# Bolstering General Practitioner Palliative Care: A Critical Review of Support Provided by Australian Guidelines for Life-Limiting Chronic Conditions

**DOI:** 10.3390/healthcare8040553

**Published:** 2020-12-11

**Authors:** Raechel A. Damarell, Deidre D. Morgan, Jennifer J. Tieman, David Healey

**Affiliations:** 1 Research Centre for Palliative Care, Death and Dying, College of Nursing and Health Sciences, Flinders University, Adelaide 5001, Australia; deidre.morgan@flinders.edu.au (D.D.M.); jennifer.tieman@flinders.edu.au (J.J.T.); 2 Palliative and Supportive Services, College of Nursing and Health Sciences, Flinders University, Adelaide 5001, Australia; dfamhealey@hotmail.com

**Keywords:** palliative care, general practitioners, practice guidelines as topic, uncertainty, professional competence, Australia, systematic review

## Abstract

General practitioners (GPs) are increasingly expected to provide palliative care as ageing populations put pressure on specialist services. Some GPs, however, cite barriers to providing this care including prognostication challenges and lack of confidence. Palliative care content within clinical practice guidelines might serve as an opportunistic source of informational support to GPs. This review analysed palliative care content within Australian guidelines for life-limiting conditions to determine the extent to which it might satisfy GPs’ stated information needs and support them to provide quality end-of-life care. Six databases and guideline repositories were searched (2011–2018). Eligible guidelines were those for a GP audience and explicitly based on an appraisal of all available evidence. Content was mapped against an established palliative care domain framework (PEPSI-COLA) and quality was assessed using AGREE-II. The nine guidelines meeting inclusion criteria were heterogenous in scope and depth of palliative care domain coverage. The ‘communication’ needs domain was best addressed while patient physical and emotional needs were variably covered. Spiritual, out-of-hours, terminal care and aftercare content was scant. Few guidelines addressed areas GPs are known to find challenging or acknowledged useful decision-support tools. A template covering important domains might reduce content variability across guidelines.

## 1. Introduction

The World Health Organisation’s Declaration of Astana calls for palliative care to be both ‘accessible to all’ and an ‘essential component of primary healthcare worldwide’ [1]. This emphasis on palliative care as a fundamental human right makes clear an expectation that palliative care should be a key responsibility of primary care clinicians, including general practitioners (GPs). In Australia, this view aligns with that of the Royal Australian College of General Practitioners (RACGP) whose standards afford ‘end-of-life care’ a central position within the GP scope of practice and the meaning of ‘comprehensive care’ [2]. GPs themselves note similarities in focus between primary and palliative care with their mutual concern for holistic, patient-centred care inclusive of the family unit [3,4].

There appears to be an expectation that GPs will increasingly provide more palliative care as the number of people reaching old age starts to exceed the capacity of specialist palliative care services [5,6,7]. Many older people will be burdened by multiple chronic comorbidities [8] requiring ongoing GP management and care coordination. Sustainable palliative care may, therefore, hinge on a ‘hierarchy of need’ model, with generalists taking on basic elements of palliative care and referring patients with more complex or refractory problems to specialist palliative care [9].

General practice palliative care offers benefits known to be valued by patients and their families, including accessibility, local knowledge, and relational continuity [10,11,12]. In Australia, where general practice operates as gatekeeper to specialist care, GPs see approximately 90% of the population annually [13]. This includes people with non-cancer life-limiting conditions such as heart failure and dementia who are often overlooked for specialist palliative care [14]. There is also growing empirical evidence that GP involvement in palliative care provides measurable benefits including improved quality of life [15,16,17], maintenance of functional status [18,19], increased likelihood of dying at home [20], and reduced health service use [16,21] with its attendant cost savings to the health system [22].

To date, there is no centralised mechanism for capturing Australian primary health care data detailing the amount of palliative care being provided by general practitioners [23,24,25]. What current information we have comes from GP self-reports. These suggest that the majority of Australian GPs are already providing some palliative care [26,27], with GPs in rural and remote areas seeing themselves as especially responsible despite being more poorly funded and resourced than their urban colleagues [28]. However, a considerable proportion of GPs (25–37%) report minimal if any interest or involvement in providing palliative care [26,29]. Given the projected population increase in the number of older people burdened by chronic diseases, high rates of GP unwillingness to care for patients with advanced-stage disease is likely to impact on the workload of other health professionals, social care services, and informal caregivers.

GPs worldwide cite similar barriers to providing palliative care for their patients such as system-level policies and processes that restrict consultation time and remuneration for more complex assessments or home visits [30,31,32]. Primary and secondary health care sector fragmentation has also created role uncertainty for GPs [15,26,33,34] and contributed to poor information flow between the various health professionals involved in care [27,35]. A substantial proportion of GPs, not only in Australia, cite a lack of confidence or skills to manage palliative care patients [36,37], which may reflect limited exposure to patients with end of life needs [4] or few training opportunities [27]. Identifying the point at which chronic or curative care should transition to palliative care [33] and communicating prognosis to patients is challenging [32]. Non-malignant conditions such as heart failure, dementia and COPD appear to be particularly problematic as their less predictable trajectories can generate prognostic uncertainty for GPs [32,38,39]. The nature of the care provided might also vary between GPs. The WHO defines the domains and goals of quality palliative care as being ‘the prevention and relief of suffering’ achieved through ‘early identification and impeccable assessment and treatment of pain and other problems, physical, psychosocial and spiritual’ [40]. While GPs express confidence in addressing patient physical suffering through pain and symptom relief [4], they appear less confident in anticipating palliative needs via assessments early in the course of a terminal illness [17,21]. Furthermore, some GPs do not promote a ‘whole-person’ view of the patient when it comes to palliative care [26], avoiding discussions of emotions and spiritual and existential concerns unless raised by patients or their family members [4,11,32,41,42]. Consequently, parts of the population may be at risk of dying without having all their palliative needs met [32,43] or the opportunity to discuss, comprehend, and plan for the end of life [10,11,44].

Australian GPs are aware of their need for additional training in palliative care and express a willingness to undertake it [26,27]. They also desire more local sources of information which they can easily absorb and apply in practice [26]. One possible source of information already available to GPs is clinical practice guidelines for individual chronic life-limiting conditions. Credible guidelines, developed via a systematic, explicit process by a panel comprising experts and representatives of all stakeholder groups, can provide sound support for clinical decision making, reduce uncertainty, and serve as a basis for communicating with patients [45,46]. Furthermore, guidelines for single life-limiting conditions such as heart failure often incorporate palliative care content in their attempt to cover the gamut of a condition’s trajectory. Guidelines might therefore play a significant role in normalising and demystifying palliative care activities for less confident GPs by specifically addressing their role within the care team, presenting evidence on the benefits of their involvement [47], describing ‘optimal’ models of integrated or shared care, and providing information on expected trajectories and signs of deterioration specific to the condition in question. Guidelines could also provide GPs with information on established decision aids, tools, strategies, and prompts designed to help them overcome some of the barriers they describe. The Supportive and Palliative Care Indicators Tool (SPICT) for early detection of palliative care needs is one example [48]. Guidelines might also underline the importance of a comprehensive and holistic patient and family assessment by organising content according to the commonly accepted biopsychosocial domains.

The extent to which guidelines intended for a GP audience currently provide support for GP palliative care is unknown. The aim of this study was therefore to evaluate the scope of the palliative care content within current high-quality Australian guidelines for chronic life-limiting illnesses using a framework comprising the domains deemed relevant to ‘best practice,’ as well as areas of practice GPs have identified as challenging. It also investigated the messages that guidelines convey, both explicit and explicit, regarding the role of the GP in providing palliative care.

## 2. Materials and Methods

This study included a systematic search for guidelines, a qualitative content analysis of guideline recommendations, and a critical appraisal of overall guideline quality.

### 2.1. Search for Guidelines

We conducted a comprehensive search for published and unpublished guidelines for chronic ‘life-limiting illnesses’, defined as conditions which might contribute directly to a person’s death [9]. The search was initially constructed and tested in OVID Medline and then translated for Ovid Embase, Joanna Briggs Institute EBP Database, PubMed, Scopus, and Web of Science. We also searched guideline repositories, organisational websites, and performed a general Google search. The search strategy comprised three components combined using AND: (1) guidelines; (2) life-limiting chronic conditions; and (3) Australia. The Ovid Medline search strategy and a list of websites targeted by the search is provided as Supplementary File S1.

### 2.2. Eligibility Criteria

Guidelines had to meet two sets of criteria:

1. Production criteria: Australian guidelines or evidence summaries, published since 2011, and reporting their methodology in full, including how the search for evidence was conducted and the basis used to formulate recommendations (e.g., consensus or evidence). The reporting criterion was important in ensuring all included guidelines were comparable in terms of their methodological and reporting quality.
2. Content criteria: Explicitly relevant to primary care, focused on the management of a chronic life-limiting condition in adults (≥18 years), and including content on palliative or end-of-life care.

Summarised versions of full guidelines, such as those published in journals, were not excluded providing they could independently meet eligibility criteria. This avoided any assumptions as to which version GPs might prefer to use. Screening proceeded in three stages. One reviewer (RD) screened all items for relevance based on their titles and abstracts alone. This was followed by a check of the full text of the remaining items against the production criteria (RD). Two reviewers (RD and DH) then independently screened the reduced set against the content criteria and discussed together any discrepancies.

### 2.3. Quality Appraisal

Two authors independently scored guideline quality using the validated AGREE-II tool [49]. This process was important for understanding each guideline’s development processes and reporting quality—two factors which might directly influence the scope and quality of the palliative care content under analysis.

### 2.4. Data Extraction

The full-text PDFs of included guidelines were imported into NVivo (QSR International). One author (RD) read full guideline content line-by-line, coding it against two pre-established, broad data nodes—one for ‘palliative/end-of-life care’ and a second for ‘communication’. The latter node was used to identify more general communication advice relevant to palliative care but not necessarily exclusive to it. Coded data were then mapped against the domains of the PEPSI-COLA palliative care framework [50], an aide memoire to guide general practitioners in conducting palliative care patient assessments. This was done to determine the nature and type of information contained within the guidelines (Table 1). These domains were therefore our a priori ‘themes’ and formed the basis of a qualitative content analysis. Guideline palliative care content not fitting into the PEPSI-COLA framework was analysed inductively and listed separately.

We chose the PEPSI-COLA tool in the absence of an existing validated framework as it was designed specifically for GPs and is intentionally holistic and comprehensive in its structure [33]. It therefore covers a wide range of palliative care activities and concerns and aligns well with the WHO definition of quality palliative care [40]. It was also developed out of the Gold Standards Framework (https://www.goldstandardsframework.org.uk/), a resource widely implemented in general practice in the United Kingdom.

## 3. Results

**Figure 1:**
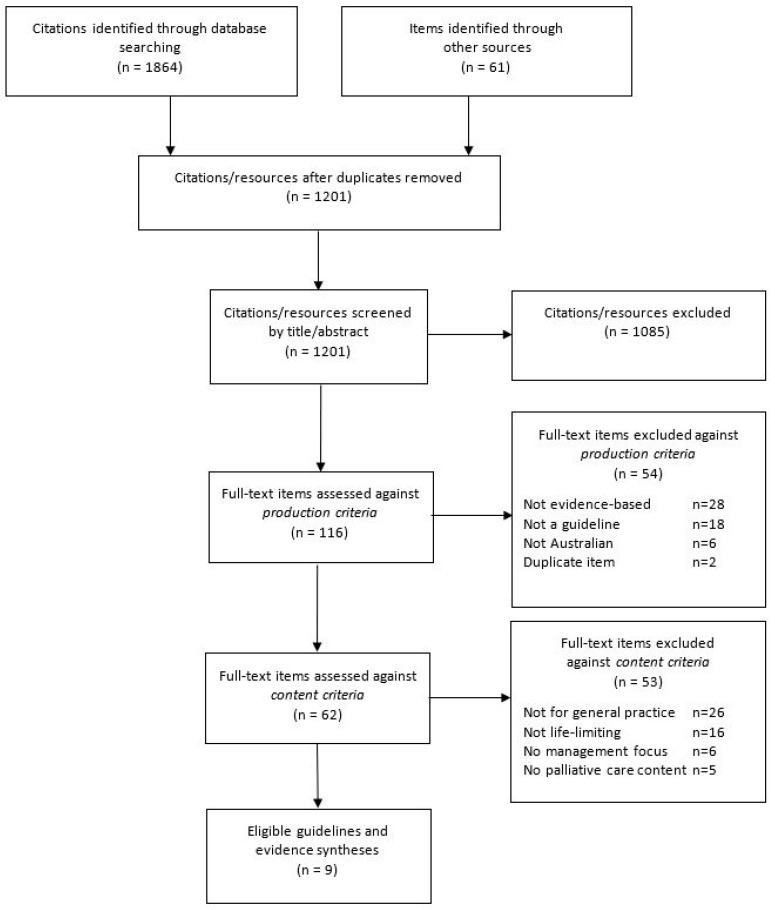
PRISMA flow diagram of the guideline selection process.

Database searches, conducted in the period 15–22 August 2018, identified 1201 unique resources. Screening based on title/abstract reduced this to 116 resources before full-text review against eligibility criteria produced a final set of nine guidance documents for life-limiting chronic conditions. The process is documented in a PRISMA flow diagram (Figure 1).

### 3.1. Characteristics of Included Guidelines

The nine resources comprised five full guidelines [51,52,53,54,55] and four summary versions of the full guidelines [56,57,58,59]. Together they covered the management of heart failure [53,56], chronic obstructive pulmonary disease [55,58,59], dementia [52,54,57], and cancer [51]. GP involvement in guideline development was noted for most guidelines. Characteristics of the nine guidelines included in this analysis are documented in Table 2.

### 3.2. Quality Appraisal

The five full guidelines were of medium to high quality, with the two dementia guidelines achieving near perfect scores. The results of the assessment are provided as Table 3.

### 3.3. Mapping of Content to PEPSI-COLA Domains

Overall, no guideline covered all domains. Most recommendations within the cancer pain management guidelines were accompanied by a statement of how the recommendation was formulated, being either evidence based or consensus based. There were only a few indications within the other guidelines as to the type of recommendation being made. These ranged in type from the weakly rated ‘[p]alliative care should include symptom control …’ [55] to the strong ‘[r]efer advanced heart failure patients to palliative care…’ [53]. Mapping of content to domains is summarised here with fuller details provided as Supplementary File S2.

#### 3.3.1. Physical Needs

None of the nine guidelines for chronic life-limiting conditions recommended a comprehensive assessment for palliative care needs in line with the WHO definition of palliative care and Australia’s National Palliative Care Standards [9], although the cancer pain guideline addressed the need to assess fully for pain at each clinical encounter, in accord with its purpose. Although all guidelines provided specific advice on symptom management in the active treatment phase, only three provided full, practical supportive care advice particular to palliative or end of life phase of care [51,52,55]. None of the three journal summary versions contained recommendations on the physical care of the palliative care patient [56,57,58]. Two guidelines [51,59] provided lists and links to tools and further resources such as a resource kit for providing culturally appropriate palliative care to Australian Aboriginal and Torres Strait Islander Peoples and lung disease specific supportive and palliative care checklists.

#### 3.3.2. Emotional Needs

Consideration for patient emotional concerns was raised by the cancer and the COPD (full) guidelines only. Advice included referring patients to a clinical psychologist for support [51] and to ‘consider discussing what death might be like’ with the patient [55]. Deprescribing guidelines alerted clinicians to the hopes and fears associated with treatments and their discontinuation [54]. Summarised versions of guidelines published in journals did not mention this aspect of care. The importance of identifying and treating depression, while covered in earlier sections of some guidelines, was not raised in the palliative care context.

#### 3.3.3. Personal Needs

Three of the nine guidelines [51,54,55] advocated the need to discuss and consider the individual patient’s values, beliefs, preferences, and experiences as well as those of their carers and families. Only the full COPD guideline mentioned spiritual or existential care as a potential need, advising clinicians to refer patients to specialist palliative care when it arose. The cancer guideline outlined the responsibility to provide culturally appropriate care and information while the full dementia guideline provided extensive coverage of cultural considerations as well as inviting concern for people with an intellectual disability, dysphagia, and low health literacy. According to the heart failure guidelines, quality of life concerns should be referred to ‘palliative care’. Guidelines summarised for journal publication did not include this aspect of care.

#### 3.3.4. Social Support Needs

Four guidelines addressed social support needs of patients. All three dementia guidelines and the full COPD guideline advised clinicians of the importance of referring patients to social support groups, advocacy services, sources of financial and legal aid, and voluntary support. These guidelines suggested that clinicians be aware of resources available locally in the community and to provide that information in written form to patients and carers.

#### 3.3.5. Information and Communication

All nine guidelines addressed this domain although each guideline had its own emphasis. The cancer guideline stressed the importance of educating patients and their families and providing verbal and written information. All three COPD guidelines focused on discussions of goals of care and formal documentation of wishes for future care, with the outcome being an ‘advanced care directive’ or ‘terminal care plan’. The three dementia guidelines centred on the need for good and clear health professional-patient communication, recommending clinicians follow specific language guidelines and undergo communication training for dementia care. Dementia guidelines also recommended that information be made available in accessible formats, calling on professional translators if necessary. Only the cancer, heart failure, and COPD (primary care) guidelines mentioned the importance of including family members and/or carers in any discussions. The dementia deprescribing guideline alone raised direct clinician-clinician liaison to ensure care continuity for the patient.

#### 3.3.6. Control and Autonomy

Seven of the nine guidelines addressed at least one aspect of patient choice, control, or autonomy such as informed or shared decision making [51,54,55], or the creation of an advance care plan [53,55,56,57,59]. The full COPD guideline discussed choice regarding place of death. Two dementia guidelines emphasised the need to ensure patient choices were explored while the person with dementia was still capable of decision making [54,57].

#### 3.3.7. Out of Hours

This domain was poorly covered by all guidelines. Only the COPD primary care guideline suggested that clinicians ‘plan for out of hours care.’ No guidelines discussed GP care for patients within the patient’s own home outside of clinic hours.

#### 3.3.8. Late (Terminal) Care

Two dementia guidelines gave specific advice on managing late stage concerns such as hydration, feeding, resuscitation, and discontinuation of medication [52,54]. The full COPD guidelines suggested that patients with challenging situations should be referred to specialist palliative care. No other guidelines addressed this domain.

#### 3.3.9. After Care

One guideline for COPD [55] gave clear advice on managing the grief and bereavement needs of family members and carers after the person’s death. The full dementia guideline, however, acknowledged that families need support ‘to deal with their grief’ [52].

### 3.4. Palliative Care Considerations Not Accommodated by PEPSI-COLA

The following aspects of palliative care were addressed in at least one guideline and may provide useful information to GPs with lower confidence in their understanding of, and skills in, palliative care.

#### 3.4.1. Defining Palliative Care

Two guidelines defined palliative care. One provided the full WHO definition [55] and the other described a ‘palliative care approach’ as the alleviation of patient symptoms, and physical, psychosocial, and spiritual needs [53].

#### 3.4.2. The GP Role

The full guideline for COPD allocated GPs a management and coordination role within a multidisciplinary care team. The COPD primary care guideline suggested that GPs develop a GP Management Plan under the Australian Medicare Benefits Scheme and arrange pharmacists to do home medicine reviews for COPD patients. The full heart failure guideline stated that GPs have a ‘vital role’ in patient management and the collaborative care model.

#### 3.4.3. Role of the Multidisciplinary Team

COPD guidelines emphasised the importance of a multidisciplinary team approach in palliative care which includes the primary care team. The heart failure guideline (full) came at this another way, addressing the specialist palliative care in advising it to work collaboratively with the patient’s GP.

#### 3.4.4. Prognostication Challenges

The full COPD guideline provided comprehensive advice on clinical indicators which might be used to initiate a review of the goals of care. It also acknowledged the challenging nature of prognosticating for COPD, suggesting palliative care initiation should not, therefore, be dependent on making an accurate diagnosis. The primary care version of the same guideline was equally as descriptive but added in the well-known ‘surprise question’ for GP consideration. The heart failure guidelines provided statistics and some general guidance on what clinicians might look for as suggestive of a change in the disease trajectory. No guideline recommended established disease-specific or generalisable tools for prognostication.

#### 3.4.5. Timing the Initiation of a Palliative Care Approach

While the COPD guidelines for primary care described anticipatory end of life care planning in detail, the two heart failure guidelines stressed the importance of involving ‘palliative care’ early in the trajectory towards end-stage heart failure.

#### 3.4.6. Benefits of Palliative Care

The COPD journal summary included a statement on the benefits of palliative care in its main recommendations.

## 4. Discussion

Clinical practice guidelines have an important educative role and, by encouraging clinicians to follow ‘best’ available practice, can build capacity and raise the quality of care [60]. This qualitative analysis of guidelines for progressive, non-curative conditions found considerable heterogeneity in the coverage of palliative care concerns across guidelines for chronic life-limiting conditions. Some of the differences inevitably and appropriately reflect condition-specific considerations, such as the relatively fuller emphasis on clinician-patient communication in the dementia guidelines. However, the rationale behind other differences, including why some domains were not addressed, is not immediately apparent. Some omissions may represent a lost opportunity to educate and guide general practitioners across the full range of considerations involved in a holistic assessment of a patient’s palliative care needs.

Despite GP uncertainty as to what constitutes palliative care [26], when it might be introduced [61], and how best to communicate a transition to the end of life phase to patients [62], most guidelines analysed did not include this foundational information. The role of the ‘impeccable assessment’ of patient needs advocated by the WHO definition of palliative care was absent from all guidelines. Furthermore, most guidelines did not mention the availability of several well-established tools for identifying palliative care needs such as question prompts (e.g., the surprise question) or practical indicator tools such as the SPICT [48].

Standard 5 of Australia’s National Palliative Care Standards stresses the importance of establishing seamless and integrated care within and between services, stating: ‘[w]hen working in partnership with other services, clear strategies for referral, communication and designated areas of responsibility are essential.’ Although the guidelines analysed originated within the same country, they provided differing advice on the timing and responsibility for referring patients to specialist care, often without clarifying explanation or mention of a needs-based assessment. One guideline simply directed clinicians to ‘refer to palliative care’ [53], while another specified referral when faced with more ‘challenging situations’ [55]. The heart failure guideline recommended clinicians involve palliative care ‘early in the heart failure trajectory’ yet elsewhere stipulated referring ‘advanced’ patients with ‘end-stage symptoms’ to palliative care [53]. The COPD guidelines supported early, anticipatory involvement of palliative care running concurrent to symptom control and active treatment [55]. All guidelines may have benefited from including a conceptual model showing how palliative care might be integrated across the varied levels of patient need, illness trajectories, and different types of palliative care providers [24].

This study confirms findings that emotional and spiritual domains are often lightly addressed, if at all, in guidelines [63]. This appears to mirror clinical practice with studies reporting psychological, social, and spiritual services infrequently deployed by GPs in end-of-life care [4,64,65,66]. We would have expected, however, more consistency in approach to recommendations across the physical domain, which has been found to be dominant in other palliative care guideline content [63]. Some guidelines provided full, symptom-specific advice for the palliative phase of care while others gave no symptom management advice, perhaps considering this aspect adequately covered in earlier chapters. The ‘information and communication’ domain was strongest across all guidelines, with most emphasising the importance of providing clear, written and culturally appropriate information to patients and their families/carers, at least on disease-specific matters. Most guidelines also advocated for exploring and documenting personal values and priorities of patients in advance care plans. Only one, however, briefly addressed communication between clinicians which is known to be a barrier to GP care provision [18]. Confirming findings of other studies, coverage was poorest for establishing care continuity out of hours [63] and considering the needs of the bereaved [65].

The GP appears as an important ‘collaborative’ team member during the active treatment phase; however, the role is less clear during the palliative care phase, varying from service coordination, symptom management, or simple referrer. Although all guidelines were situated within the same national context, no guideline addressed the importance of having an effective continuum of palliative care within the Australian health care system which includes the general practitioner. Until the GP role in palliative care is clarified along with the domains and processes that fall within the GP’s remit, there seems little benefit to discussions of how to assess GP competence in providing palliative care [67]. This has important implications for the safety, quality and equity of care currently being provided by general practice. Although guidelines cannot resolve concerns with service delivery mechanisms and settings, as these will be influenced by numerous local and systemic factors, recognising these constraints is important given the evidence of GPs’ self-identified needs relating to palliative care [4,18,30,32,37]. The fact that these areas are not addressed across the guidelines analysed could be seen as a lost opportunity if non-specialist palliative care is deemed a policy priority and expectation and yet a significant proportion of GPs lack the confidence, education, or skills to provide it. These findings also run contra to World Health Assembly’s recommendation that member states ‘develop and strengthen … evidence-based guidelines …that adequately address ethical issues related to the provision of comprehensive palliative care, such as equitable access, person-centred and respectful care … and to inform education in pain and symptom management and psychosocial support’ [68].

Overall, there was considerable heterogeneity across guidelines as to domains covered and domain elements emphasised. Despite access to the same evidence base, no two guidelines made similar recommendations on the same aspect of care, differing in content, scope and directiveness. Summary versions of full guidelines also differed in the amount and type of information they conveyed, generally omitting much of the palliative care content. Whilst space constraints make this understandable, this still matters if time-poor GPs rely on shorter, more readily accessible guidelines rather than the considerably more informative full versions. Despite being the longest of the guideline summaries, the COPD concise guide for primary care [59] combines a visually interesting layout with concise information, effectively highlighting evidence-based recommendations while providing abundant, yet discreetly placed, practice tips, links to useful supplementary resources, and contact information for relevant local community resources such as the Quitline for smokers. This linking of the evidence of *what to do* with practical resources advising *how to do it* arguably provides GPs with more useful guidance than long resources focused purely on providing a precise appraisal of published research studies.

In crafting end-of-life guidance, guideline developers and their authorship teams have available to them a considerable body of evidence describing the barriers faced by GPs in providing palliative care to their patients. They might then proactively address those barriers relating to skills, confidence, and knowledge by providing information and guidance in the areas where GPs express uncertainty such as prognostication and timing of referral. General practitioners are already well suited to provide palliative care. They have the benefit of being able to draw on knowledge of a patient and his/her family gained through relational and longitudinal care continuity. Their holistic, biopsychosocial understanding of the illness experience also fits well with the goals and ethos of palliative care. Guidelines might highlight these strengths alongside others to bolster GP confidence in providing palliative care. Furthermore, in clarifying the GP’s role and the complementary roles played by other health care professionals, they might also describe an integrated system where the various players work together, rather than on their own, to create seamless care experiences for families.

Incorporating more of the domains closely associated with palliative care might also strengthen the quality of palliative care guideline content for general practitioners. This could be achieved by developing a template for palliative care content based on discussion and consensus between stakeholders including guideline developers, GPs, medical specialists, members of specialist palliative care teams, as well as patients, and their family/carers. This template could be adaptable to fit any guideline development project with scope to customise for disease-specific factors. This has been done previously with the creation of template that intercalates palliative care considerations across the full range of the guideline, rather than leaving it until the end of the document [69].

This study is based on a comprehensive search for and analysis of guidelines in one national context only. However, given anticipated increases in ageing populations, multimorbidity, and expected deaths worldwide, it may be timely for international guideline groups to consider the strength and utility of the palliative content within their own guidelines.

This study took an objective approach to analysing the scope and potential utility of palliative care guidance by adopting an *a priori* framework commonly used within palliative care to assess individual patient needs. The analysis was also situated within the context of two existing discourses on the topic of GP involvement in palliative care provision: (1) the expectations of prominent health organisations such as WHO; and (2) GP reports on their own levels of confidence and engagement.

This study does make several basic assumptions. Firstly, that single disease guidelines are the appropriate place for guidance on palliative care, especially patient-centred aspects such as communication which may not be supported by strong empirical evidence. Perhaps referencing standalone palliative care guidelines, where they exist, is a more effective way to ensure that this important aspect of care is adequately covered. Development of these guidelines might be based on different evidentiary and knowledge foundations than disease-specific ones, offering more flexibility in the range of topics they can cover. The palliative care guidance in the RACGP Aged Care Clinical Guide is an example of this approach [70].

Another assumption is that the PEPSI-COLA domains adequately reflect the aspects of care GPs are happy to provide and which patients feel comfortable in receiving. Spiritual care is a case in point. Patients are often not aware that this might be a topic they can raise with their GP [11]. Perhaps the most interesting assumption we have made is that it is incumbent on GPs to be providing palliative care at all, certainly without having a full understanding of the local organisational strictures they work within such as the combined pressures of time, patient complexity, and remuneration systems which lack incentives for providing care outside of the clinic and regular work hours. Finally, this study did not ask GPs for their views on the adequacy of guideline palliative care content for their own practice. We hope this might form the basis of a future investigation.

## Figures and Tables

**Table 1:** PEPSI-COLA aide memoire for holistic palliative care patient assessment [50].

Domain of Need	Considerations
Physical	Physical needs including symptom control and prevention and/or relief from medication side effects.
Emotional	Emotional needs including psychological assessment, understanding patient wishes for information, mood, anxiety, coping, and fears.
Personal	Personal needs including cultural, language, religious, or spiritual needs.
Social support	Social care needs of patient and carer(s). Includes practical concerns such as managing at home and at work, financial concerns, family and close relationships, social life and recreation, and concern for dependents.
Information communication	Information and communication needs within the health care team: between clinicians, to and from patient, and to and from carers.
Control and autonomy	This includes assessing mental capacity to make decisions around choice, determining the person’s preferences for treatment options, and advance care planning.
Out of hours and emergency	Identifying and establishing contacts for ensuring continuity of care after-hours. This includes informing patient and family of arrangements, letting after-hours GPs/locum services know of patient’s needs, and ensuring patients and carers have access to medications and equipment for when required.
Late care	Care considerations at the very end of life. This might include stopping non-urgent medications, communicating stage of condition to patient and family, alerting them to what might happen (e.g., rattle and agitation), providing comfort measures, and death pronouncement.
After care	Bereavement needs including bereavement risk assessment and follow up with the family.

**Table 2:** Characteristics of included guidelines.

Condition: Specific Topic (Date)	Development Organisation	Description	Length in Pages	GP Involvement in Guideline Production
Cancer: pain management (2013)	Australian Adult Cancer Pain Management Guideline Working Party and Cancer Council Australia	Full guideline (adapted)	NA (Online)	No
Chronic obstructive pulmonary disease (2018)	Lung Foundation Australia and the Thoracic Society of Australia and New Zealand	Full guideline (update)	210	Yes
Chronic obstructive pulmonary disease (2017a)	Lung Foundation Australia and the Thoracic Society of Australia and New Zealand	Guideline summary	7	Yes
Chronic obstructive pulmonary disease: primary care (2017b)	Lung Foundation Australia, the Thoracic Society of Australia and New Zealand, and The Royal Australian College of General Practitioners	Guideline summary for primary care	40	Yes
* Dementia: deprescribing cholinesterase inhibitors and memantine (2018)	University of Sydney, Cognitive Decline Partnership Centre, and Bruyère Research Institute	Full guideline (new)	132	Yes
* Dementia (2016)	Cognitive Decline Partnership Centre. Guideline Adaptation Committee	Full guideline (adapted)	136	Yes
* Dementia (2016)	Cognitive Decline Partnership Centre. Guideline Adaptation Committee	Guideline summary	6	Yes
Heart failure (2018)	National Heart Foundation of Australia and Cardiac Society of Australia and New Zealand	Full guideline (update)	86	Yes
Heart failure (2018)	National Heart Foundation of Australia and Cardiac Society of Australia and New Zealand	Guideline summary	7	Yes

* Guidelines have been endorsed for quality by the Australian Government’s National Health and Medical Research Council.

**Table 3:** AGREE II scores per domain for individual guidelines.

Guideline	Scope and Purpose (%)	Stakeholder Involvement (%)	Rigor of Development (%)	Clarity and Presentation (%)	Applicability (%)	Editorial Independence (%)	Average Score (%)
Heart failure							
Full guideline	86.1	47.2	72.9	100	68.8	50.0	70.8
Chronic Obstructive Pulmonary Disease (COPD)							
Full guideline	80.6	88.9	69.8	88.9	37.5	100	77.6
Dementia							
Full guideline	100.0	100.0	100.0	100.0	79.2	100.0	96.5
Deprescribing cholinesterase inhibitors and memantine	97.2	100.0	96.9	91.7	88.3	100.0	95.7
Cancer							
Cancer pain management in adults	75.0	75.0	78.0	97.0	37.5	61.9	70.7
Average standardised domain score	88.9	82.4	85.2	95.8	64.4	81.9	83.1

## Supplementary Materials

The following are available online at https://www.mdpi.com/2227-9032/8/4/553/s1, Table S1: Ovid MEDLINE search strategy, Table S2: Directive guideline content mapped to PEPSI-COLA framework, Table S3: Other considerations not accommodated by PEPSI-COLA framework.
