# Supplementary material for: Bolstering General Practitioner Palliative Care: A Critical Review of Support Provided by Australian Guidelines for Life-Limiting Chronic Conditions
Source: Healthcare (Basel). 2020 Dec 11;8(4):553. doi: 10.3390/healthcare8040553 (PMC7763828; doi:10.3390/healthcare8040553)
Supplement: Supplementary file 1 [file healthcare-08-00553-s001.zip › healthcare-1002990-Supplementary file S1.pdf]

healthcare

MDPI

SUPPLEMENTARY FILE S1. SEARCH STRATEGIES FOR IDENTIFYING GUIDELINES

Table S1. Ovid MEDLINE search strategy

#	Searches
1	Critical Pathways/
2	Clinical Protocols/
3	Consensus/
4	consensus development conference/ or consensus development conference, nih/
5	consensus development conferences as topic/ or consensus development conferences, nih as topic/
6	guideline/ or practice guideline/
7	guidelines as topic/ or practice guidelines as topic/
8	Health Planning Guidelines/
9	(consensus development conference or consensus development conference, nih or guideline or practice guideline).pt.
10	(position statement* or policy statement* or practice parameter* or best practice*).ti,kf,kw.
11	(standards or guideline or guidelines).ti,kf,kw.
12	((practice or treatment* or clinical) adj guideline*).ab.
13	(CPG or CPGs).ti.
14	consensus*.ti,kf,kw.
15	consensus*.ab. /freq=2
16	((critical or clinical or practice) adj2 (path or paths or pathway or pathways or protocol*)).ti,kf,kw.
17	recommendat*.ti,kf,kw.
18	(care adj2 (standard or path or paths or pathway or pathways or map or maps or plan or plans)).ti,kf,kw.
19	(algorithm* adj2 (pharmacotherap* or therap* or treatment* or intervention*)).ti,kf,kw.
20	or/1-19
21	exp *neoplasms/

22	(Cancer* or melanoma* or myeloma* or sarcoma* or lymphoma* or neuroblastoma* or retinoblastoma* or osteosarcoma* or tumor* or tumour* or malignan* or neoplas* or leukemi* or leukaemi* or carcinoma* or adenocarcinoma*).ti.
23	exp *Coronary Disease/
24	((Coronary or ischemi* or ischaemi*) adj2 disease*).ti.
25	exp *Dementia/ or (dementia* or Alzheimer* or frontotemporal lobar degeneration* or pick disease* or picks disease*).ti.
26	*cardiovascular diseases/ or cardiovascular disease*.ti.
27	*Myocardial Ischemia/ or *Heart diseases/
28	((myocardial or heart) adj (disease* or ischemi* or ischaemi*)).ti.
29	exp *cardiomyopathies/ or exp *heart failure/
30	(cardiomyopath* or heart failure).ti.
31	*Pulmonary Disease, Chronic Obstructive/ or (chronic obstructive pulmonary disease* or COPD).ti.
32	*renal insufficiency, chronic/ or *kidney failure, chronic/
33	(Chronic adj (renal or kidney)).ti.
34	*liver failure/ or *end stage liver disease/
35	((liver or hepatic) adj (failure or disease*)).ti.
36	(chronic adj (liver or hepatic)).ti.
37	*neurodegenerative diseases/ or *lewy body disease/ or *motor neuron disease/ or *amyotrophic lateral sclerosis/ or *bulbar palsy, progressive/ or *muscular atrophy, spinal/ or *parkinson disease/
38	(neurodegenerative or lewy body or motor neuron* or amyotrophic lateral sclerosis or progressive bulbar palsy or parkinson* or (MND or ALS)).ti.
39	*multiple sclerosis/ or *multiple sclerosis, chronic progressive/ or *multiple sclerosis, relapsing-remitting/
40	multiple sclerosis.ti.
41	or/21-40
42	exp Australia/ or (australia* or australasia* or new south wales* or victoria* or queensland* or tasmania* or northern territory*).ti,ab,kw,kf.
43	20 and 41 and 42
44	limit 43 to yr="2012 -Current"
45	limit 44 to english language

Notes:

Includes database subsets: Ovid Medline (1946 to August 03, 2018); Epub Ahead of Print, In-Process & Other Non-Indexed Citations; Daily; and Versions.

Lines 1-19 represent the ‘guidelines’ part of the search. This is based on an existing search by Canadian Agency for Drugs and Technologies in Health (CADTH).
/ = Medical Subject Heading (MeSH) search; ti = title field search; ab = abstract field search; kw = exact author keyword field search; kf = author keyword field search; pt = publication type field search; adj2 = search words can occur within two spaces of each other, in either order; ab. /freq=2 = words must appear with a frequency of 2 (i.e. twice) within the abstract field.

Sources used to identify Australian clinical practice guidelines

Guideline repositories and sources of evidence summaries

- Clinical Practice Guidelines Portal (Australia): https://www.clinicalguidelines.gov.au/portal
- Guidelines International Network (GIN): http://www.g-i-n.net/
  MJA's 'Guidelines and Statements' section: https://www.mja.com.au/journal/guidelines
- eTG Complete (subscription resource)
- Australian Medicines Handbook (subscription resource)
- Handbook of Non-Drug Interventions: HANDI: https://www.racgp.org.au/clinical-resources/clinical-guidelines/handi

Medical speciality colleges or societies, or organisations

- Royal Australian College of General Practitioners (RACGP): https://www.racgp.org.au/
- Royal Australian College of Physicians (RACP): https://www.racp.edu.au/
- Centre for Remote Health: https://www.crh.org.au/remote-phc-manuals-overview
  - CARPA Standard Treatment Manual
  - Minymaku Kutju Tjukurpa: Women's Business Manual
- Australian Primary Health Care Research Institute: https://rsph.anu.edu.au/research/centres-departments/australian-primary-health-care-research-institute
- AusDoc.Plus 'Guide to Guidelines': https://www.ausdoc.com.au/guidetoguidelines

Palliative care organisational websites

- Palliative Care Australia (PCA): https://palliativecare.org.au/
- CareSearch: https://www.caresearch.com.au/Caresearch/Default.aspx
- End of Life Directions for Aged Care (ELDAC): https://www.eldac.com.au/
- PalliAGED: https://www.palliaged.com.au/

Condition-specific organisational websites

- Cancer Council Australia (CCA): https://wiki.cancer.org.au/australia/Guidelines
- Cancer Australia: https://canceraustralia.gov.au/
- Clinical Oncological Society of Australia (COSA): https://www.cosa.org.au/
- Australian Heart Foundation: https://www.heartfoundation.org.au/
- Cardiac Society of Australia and New Zealand: http://www.csanz.edu.au/
- Lung Foundation Australia: https://lungfoundation.com.au/
- Thoracic Society of Australia and New Zealand: https://www.thoracic.org.au/

- Kidney Health Australia: http://kidney.org.au/
- Gastroenterological Society of Australia (GESA): http://www.gesa.org.au/
- Australian Liver Foundation: http://liver.org.au/
- Dementia Australia: https://www.dementia.org.au/
- MND Australia: https://www.mndaust.asn.au/
- Parkinson's Australia: https://www.parkinsons.org.au/
- MS Australia: https://www.msaustralia.org.au/

Government sites

- Australian Government Department of Health: https://www.health.gov.au/
- National Health and Medical Research Committee (NHMRC): https://www.nhmrc.gov.a
