# Supplementary material for: Bolstering General Practitioner Palliative Care: A Critical Review of Support Provided by Australian Guidelines for Life-Limiting Chronic Conditions
Source: Healthcare (Basel). 2020 Dec 11;8(4):553. doi: 10.3390/healthcare8040553 (PMC7763828; doi:10.3390/healthcare8040553)
Supplement: Supplementary file 1 [file healthcare-08-00553-s001.zip › healthcare-1002990-Supplementary file S2.pdf]

healthcare

MDPI

# SUPPLEMENTARY FILE S2

Table S2. Directive guideline content mapped to the PEPSI-COLA framework

| | | | | |
|---|---|---|---|---|
| **Physical** | **Cancer: pain management** | Assess pain intensity… (Evidence Based Recommendation) | Complete a comprehensive assessment if … (Evidence Based Recommendation) | Pharmacological and non-pharmacological advice for managing pain (Evidence Based Recommendation) |
| | **COPD (full)** | Palliative care … should include symptom control (Level II, weak) | Provides specific pharmacological and non-pharmacological advice on managing breathlessness | Refer patients with more challenging situations to specialist palliative care for management of persisting refractory symptoms |
| | **COPD (journal summary)** | | | |
| | **COPD (primary care summary)** | Proactive management of symptoms like chronic breathlessness, and treatments for likely severe complications like panic for severe dyspnoea | | |
| | **Dementia (full)** | Covers palliative management of fever, pain, agitation, shortness of breath | Hydration, feeding, symptom management and the prescription of medications | |
| | **Dementia (journal summary)** | | | |

| | | | | |
|---|---|---|---|---|
| | **Dementia: deprescribing** | Discontinuation recommended if cognition and/or function has significantly worsened over the past six months (or less, as per the individual) | | |
| | **Heart failure (full)** | Refer advanced HF patients to palliative care to alleviate end-stage symptoms (Strong recommendation FOR, high quality evidence) | | |
| | **Heart failure (summary)** | Refer advanced HF patients to palliative care to alleviate end-stage symptoms (GRADE: Strong; Evidence: High) | | |
| **Emotional** | **Cancer: pain management** | Provide support for any psychosocial and spiritual concerns identified during comprehensive assessment (Evidence Based Recommendation) | Consider referral to a clinical psychologist for psychological therapies and support (Evidence Based Recommendation) | |
| | **COPD (full)** | Palliative care … should address psychosocial issues (Level II, weak) | Refer patients with more challenging situations to specialist palliative care for psychosocial care | Consider discussing what death might be like |
| | **COPD (journal summary)** | | | |
| | **COPD (primary care summary)** | | | |

| | | | | |
|---|---|---|---|---|
| | **Dementia (full)** | | | |
| | **Dementia (journal summary** | | | |
| | **Dementia: deprescribing** | Deprescribing recommendations can be complicated by life-limiting nature of dementia…with significant hope being placed in these medications by people with dementia and their family | Carers have expressed fears associated with medication discontinuation, and individuals may feel that deprescribing is 'giving up' or a signal that they are no longer worth treating. | |
| | **Heart failure (full)** | | | |
| | **Heart failure (summary)** | | | |
| **Personal** | **Cancer: pain management** | Adopt a person-centred approach to pain management<br>(Evidence Based Recommendation) | Take into account the patient's needs and preferences<br>(Evidence Based Recommendation) | Provide culturally appropriate care and information<br>(Evidence Based Recommendation) |
| | **COPD (full)** | Refer patients with more challenging situations to specialist palliative care for spiritual or existential care | Consider discussing patients' and carers' values and beliefs | Clinical support teams working with the primary healthcare team can enhance quality of life and reduce disability for patients with COPD<br>(Level III-2, weak recommendation) |
| | **COPD (journal summary)** | | | |

| | | | | |
|---|---|---|---|---|
| | **COPD (primary care summary)** | | | |
| | **Dementia (full)** | Consider:<br>- health literacy<br>- the specific needs of people with dysphasia or an intellectual disability<br>- Indigenous Australians<br>- Culturally and linguistically diverse people (CALD) | | |
| | **Dementia (journal summary)** | | | |
| | **Dementia: deprescribing** | Consider the values, preferences and experiences of the person with dementia and/or their carer/family when determining if trial deprescribing is appropriate. | Any discussion about values and preferences with regard to ChEI and/or memantine therapy must consider the expectations of people with dementia and their carer/family in regard to therapy benefit | |
| | **Heart failure (full)** | Refer advanced HF patients to palliative care to improve quality of life, [and] decrease rehospitalisation<br>(Strong recommendation FOR, high quality evidence) | | |

| | | | | |
|---|---|---|---|---|
| | **Heart failure (summary)** | Refer to palliative care to improve quality of life and decrease rehospitalisation. (GRADE: Strong; Evidence: High) | | |
| **Social support** | **Cancer: pain management** | | | |
| | **COPD (full)** | | | |
| | **COPD (journal summary)** | | | |
| | **COPD (primary care summary)** | Refer patients to the Lung Foundation to be put into contact with patient support groups and educational resources | | |
| | **Dementia (full)** | Inform the person with dementia, their carer(s) and family about advocacy services, financial and legal advice, and voluntary support | Provide information on how to join a social support group | Written and verbal information about services available in the community |
| | **Dementia (journal summary)** | Provide information for the person and their carer(s) and families regarding sources of financial and legal advice, advocacy; and how to join a support group (Consensus Based Recommendation) | Provide written and verbal information regarding Alzheimer's Australia, Carers Australia, Aged Care Assessment Teams and My Aged Care (Practice point) | Provide information in a verbal and written form on community services, and record the advice and information given |

| | | | | |
|---|---|---|---|---|
| | **Dementia: deprescribing** | The primary care physician or family physician should be aware of the resources available in their local area to support people with dementia and their carers. | | |
| | **Heart failure (full)** | | | |
| | **Heart failure (summary)** | | | |
| **Information & communication** | **Cancer: pain management** | Provide patients with education about cancer-related pain and its management. (Evidence Based Recommendation) | Provide patients with verbal and written information on pain and its management (Evidence Based Recommendation) | Include the person's family, carers and significant others in education about pain and its management, if appropriate. (Consensus Based Recommendation) |
| | **COPD (full)** | Discussing goals of care and future treatment wishes should occur early, in a non-acute setting and should involve their General Practitioner.<br><br>Routinely ask if patients wish to discuss or update their goals of care | Terminal care plans to be documented and communicated to all services involved in the care of the patient for continuity of care | Introduce discussions over multiple appointments… gently adding each new topic gradually … reducing the chance of causing distress |
| | **COPD (journal summary)** | A plan of care should be developed with the multidisciplinary team. | Discussion regarding advanced<br>care directives should be undertaken as part of usual management<br>at a suitable time in the disease course. | |

| | | | | |
|---|---|---|---|---|
| | **COPD (primary care summary)** | Encourage all patients to involve carers and family members in their management (e.g. by attending consultations) | Initiate discussion about possible future care requirements with the patient to understand their wishes. | |
| | **Dementia (full)** | Use language that is consistent with the Dementia Language Guidelines and the "Talk to me" good communication guide for talking to people with dementia.<br><br>Be honest and respectful and use a gradual and individualised approach when communicating the diagnosis to the person with dementia and their carer(s) and family.<br><br>Health professionals should be trained in communicating clearly with the person with dementia, their carer(s) and family and to provide person-centred care<br><br>The health professional should convey to the family when palliative care is indicated, why it is recommended and what is involved. | Provide information in the preferred language and an accessible format.<br><br>Provide professional translators or cultural interpreters | CALD carers and families should receive support, education, and information. |

| | | | | |
|---|---|---|---|---|
| | **Dementia (journal summary** | Follow the 'Talk to me' good communication guide and Alzheimer's Australia Dementia language guidelines when communicating with people with dementia. | Information should be provided for the person and their carer(s) and families regarding: the signs and symptoms of dementia; the course and prognosis of the condition; and treatments<br>(Consensus Based Recommendation) | Use an individual, gradual, respectful, and honest approach when communicating the diagnosis.<br><br>After diagnosis, provide person with dementia, carer(s), and family with accessible information in verbal and written forms about dementia signs and symptoms, course and prognosis, and treatments |
| | **Dementia: deprescribing** | Consider the viewpoints of the person with dementia and their carers and include education about the potential benefits versus harms of both continuing and discontinuing the medications.<br><br>The individual and/or carer/family should be aware of what to look out for and what to do if a change in condition occurs—consider verbal and written communication. | Good communication between clinicians and people with dementia and/or carers/family about the benefits and harms of continuing versus discontinuing, in the context of their values and preferences, is necessary | Other healthcare professionals may need to be consulted to determine the appropriateness to trial withdrawal, or to ensure monitoring is conducted throughout the process.<br><br>Liaison with other healthcare professionals … e.g. the community pharmacist may need to make alterations to dosage administration aids |
| | **Heart failure (full)** | Shift to a palliative care focus should be discussed with patient, family, cardiologist … and GP (Rationale) | Discussions concerning ICD deactivation should involve …<br>(Practice advice) | |

| | | | | |
|---|---|---|---|---|
| | **Heart failure (summary)** | Discussions concerning ICD deactivation should occur between the patient, family, and cardiologist (Practice point) | | |
| **Control** | **Cancer: pain management** | Adopt a person-centred approach to pain management which involves enabling the person to make informed decisions about their care and treatment (Evidence Based Recommendation) | | |
| | **COPD (full)** | Terminal care plans, advanced directive, location of care to be documented | Consider discussing treatment options including … admission to intensive care unit … [and] end-of-life care wishes, including place of death preferences | |
| | **COPD (journal summary)** | | | |
| | **COPD (primary care summary)** | Anticipatory care planning includes advance care planning and ensuring there is a substitute decision maker | | |
| | **Dementia (full)** | | | |
| | **Dementia (journal summary)** | Discuss the use of an enduring guardianship, enduring power of attorney and advance care plans with patients with dementia, their carer(s) and | | |

| | | | | |
|---|---|---|---|---|
| | | family while they have capacity (Practice point). | | |
| | **Dementia: deprescribing** | Decisions surrounding deprescribing should be conducted as shared decision making with the person with dementia and/or their family/carer | Discussions about dementia medication prescribing and eventual decisions about deprescribing should occur early in therapy, when the person with dementia is still able to participate in decision making | The person with dementia should be included in the conversation |
| | **Heart failure (full)** | Patients with heart failure should be encouraged to have an advanced care plan, regardless of clinical status and soon after diagnosis | | |
| | **Heart failure (summary)** | Patients with heart failure should be encouraged to have an advanced care plan, regardless of clinical status and soon after diagnosis (Practice point) | | |
| **Out of hours** | **Cancer: pain management** | | | |
| | **COPD (full)** | | | |
| | **COPD (journal summary)** | | | |

| | | | | |
|---|---|---|---|---|
| | **COPD (primary care summary)** | Plans for out of hours care | | |
| | **Dementia (full)** | | | |
| | **Dementia (journal summary)** | | | |
| | **Dementia: deprescribing** | | | |
| | **Heart failure (full)** | | | |
| | **Heart failure (summary)** | | | |
| **Late** | **Cancer: pain management** | | | |
| | **COPD (full)** | Refer patients with challenging situations to specialist palliative care for active management of the terminal phase (at home or in a hospice) | Hospice or specialist consultations should be available if required | Terminal care plans may be appropriate for patients who elect to avoid active management. |
| | **COPD (journal summary)** | | | |
| | **COPD (primary care summary)** | | | |

| | | | | |
|---|---|---|---|---|
| | **Dementia (full)** | Any decision about rehydration should be made in conjunction with the carer(s) and family after providing them with up-to-date information on the potential benefits and harm. | In the absence of a valid and applicable advance directive to refuse resuscitation, the decision to resuscitate should take account of any expressed wishes or beliefs of the person with dementia, together with the views of the carer(s) and family and the multidisciplinary team. | Specific decisions may need to be made (by proxy decision-makers) regarding hydration, feeding, symptom management and the prescription of medications. |
| | **Dementia (journal summary)** | | | |
| | **Dementia: deprescribing** | Discontinuation recommended if the individual has severe/end-stage dementia or non-dementia terminal illness | We recommend trial discontinuation if the individual has severe/end-stage dementia (some characteristics of this stage include dependence in most activities of daily living, inability to respond to their environment and/or limited life expectancy) | |
| | **Heart failure (full)** | | | |
| | **Heart failure (summary)** | | | |
| **After care** | **Cancer: pain management** | | | |

| | | | | |
|---|---|---|---|---|
| | **COPD (full)** | Refer to specialist palliative care for emotional care and bereavement support of relatives and carers | | |
| | **COPD (journal summary)** | | | |
| | **COPD (primary care summary)** | | | |
| | **Dementia (full)** | Families need support to help them in their role as proxy decision-makers and to deal with their grief. | | |
| | **Dementia (journal summary)** | | | |
| | **Dementia: deprescribing** | | | |
| | **Heart failure (full)** | | | |
| | **Heart failure (summary)** | | | |

Table S3. Other considerations not accommodated by PEPSI-COLA framework

| | | | | |
|---|---|---|---|---|
| **Definition of palliative care** | **Cancer: pain management** | | | |
| | **COPD (full)** | Provides WHO definition (2002) | | |
| | **COPD (journal summary)** | | | |
| | **COPD (primary care summary)** | | | |
| | **Dementia (full)** | | | |
| | **Dementia (journal summary)** | | | |
| | **Dementia: deprescribing** | | | |
| | **Heart failure (full)** | The palliative care approach focuses on alleviation of symptoms and the patient's physical, psychosocial and spiritual needs. (Rationale) | | |
| | **Heart failure (summary)** | | | |
| **GP role** | **Cancer: pain management** | | | |

| | | | | |
|---|---|---|---|---|
| | **COPD (full)** | General palliative care practices such as symptom management and aligning treatment with patients' goals should be routine aspects of care. | As the primary healthcare provider, the general practitioner (GP) is uniquely placed to identify smokers and help them quit, diagnose COPD in its early stages and coordinate care as the disease progresses. | Mild to moderate COPD: GPs will manage patients, and coordinate investigation and management<br><br>Advanced COPD: GPs play a crucial role coordinating services provided by a range of healthcare professionals and care agencies (the "multidisciplinary team"). |
| | **COPD (journal summary)** | | | |
| | **COPD (primary care summary)** | Consider developing a GP Management Plan (GPMP, Item 721) and a Team Care Arrangement (TCA, Item 723) in addition to organising a home medicines review with a pharmacist. | Re-assess patients at least annually and 3 weeks after each exacerbation. | |
| | **Dementia (full)** | | | |
| | **Dementia (journal summary)** | | | |
| | **Dementia: deprescribing** | The primary care physician or family physician should be aware of the resources available in their local area to support people with dementia and their carers | | |

| | | | | |
|---|---|---|---|---|
| | **Heart failure (full)** | The benefits of a collaborative care model between GPs or general physicians with involvement from cardiologists should be considered. | GPs have a vital role in the management of patients with heart failure in the community. | Systems of care for heart failure usually include a multidisciplinary heart failure specialist team and the patient's GP. |
| | **Heart failure (summary)** | | | |
| **The role of the multidisciplinary team** | **Cancer: pain management** | Routinely establish a multidisciplinary team approach to pain management (Evidence Based Recommendation) | | |
| | **COPD (full)** | Palliative care - ideally from a multidisciplinary team which includes the primary care team (Level II, weak) | Terminal care does not always require specialist palliative care. | Active treatment of persisting symptoms or challenging issues may require a multidisciplinary team (which includes primary care, respiratory medicine, and palliative care)<br><br>Patients and their family and friends should be actively involved in a therapeutic partnership with a range of health professionals. |
| | **COPD (journal summary)** | A plan of care should be developed with the multidisciplinary team. | | |
| | **COPD (primary care summary)** | Some evidence suggests that multidisciplinary teams (where available) assisting GPs can safely and successfully treat carefully selected | | |

| | | | | |
|---|---|---|---|---|
| | | patients with COPD presenting with exacerbations of COPD, at home with support from respiratory nurses. | | |
| | **Dementia (full)** | | | |
| | **Dementia (journal summary)** | | | |
| | **Dementia: deprescribing** | | | |
| | **Heart failure (full)** | The palliative care service should work collaboratively with the patient's heart failure team and GP. (Practice advice) | | |
| | **Heart failure (summary)** | | | |
| | **Cancer: pain management** | | | |
| | **COPD (full)** | Functional deterioration in the presence of optimum treatment requires a reappraisal of the goals of care. | Each exacerbation may be reversible until there is a suboptimal or no response to treatment. At this point the patient may enter their terminal phase and the goals of care may change rapidly to palliation with treatment limitations or palliation alone with withdrawal of active therapy. | Accurate assessment of approaching end of life is difficult.<br><br>Good chronic disease care involves considering if the person is near the end of life and planning accordingly. |

| | | | | |
|---|---|---|---|---|
| | | | | Given the difficulty in determining prognosis in an individual with COPD, including palliative care principles and practices into COPD management should not be dependent on making an accurate prognosis. |
| **Prognostication (e.g. information, indicators, tools)** | **COPD (journal summary)** | | | |
| | **COPD (primary care summary)** | Anticipatory care planning appropriate when the patient is severely symptomatic or has had multiple exacerbations in the last 12 months.<br><br>Includes the 'surprise question'. | | |
| | **Dementia (full)** | The prognosis for people with dementia is often unclear and clinicians may be reluctant or unable to provide a clear prognosis. | | |
| | **Dementia (journal summary)** | The duration from diagnosis of dementia to death is highly variable with a review finding that the average duration across studies ranged from 1.1 to 8.5 years. | | |
| | **Dementia: deprescribing** | | | |

| | | | | |
|---|---|---|---|---|
| | **Heart failure (full)** | Nearly 40% of patients diagnosed with heart failure will die within 12 months of their first hospitalisation for heart failure. | As their heart failure progresses towards end-stage, patients begin to experience diverse debilitating symptoms, increasing the distress of both the patient and their carers, particularly during their last 6 months of life. | Although the evidence is limited, early post-hospital discharge appointments should be considered to identify potential issues or signs and symptoms that may indicate early exacerbation of heart failure. |
| | **Heart failure (summary)** | | | |
| | **Cancer: pain management** | | | |
| | **COPD (full)** | Concurrent symptom control, palliative care approach and active treatment. | Palliative care should be considered early. | |
| | **COPD (journal summary)** | Discussion regarding advanced care directives should be undertaken as part of usual management at a suitable time in the disease course. | | |
| | **COPD (primary care summary)** | Anticipatory end of life planning described in detail. | | |
| | **Dementia (full)** | It is important that health professionals are honest and truthful when communicating the diagnosis to the person with dementia and those close to them. How and when that occurs must be managed with sensitivity to the person | | |

| | | | | |
|---|---|---|---|---|
| **Timing (e.g. early identification of needs, early involvement of palliative care services)** | | with dementia's wishes, their relationship with the medical practitioner providing the diagnosis and the context of the discussion. | | |
| | **Dementia (journal summary)** | | | |
| | **Dementia: deprescribing** | | | |
| | **Heart failure (full)** | It is important to have palliative care involvement early in the heart failure trajectory to reduce the suffering and distress associated with these symptoms and a terminal condition. | Involvement of palliative care should be considered early in the trajectory towards end-stage heart failure. (Strong recommendation FOR, high quality evidence) | Ideally, referrals to a palliative care service should be implemented early in patients with advanced heart failure. |
| | **Heart failure (summary)** | Involvement of palliative care should be considered early in the trajectory towards end-stage HF. (GRADE: Strong. Evidence: High) | | |
| **Benefits of palliative care** | **Cancer: pain management** | | | |
| | **COPD (full)** | Early palliative care provision can improve survival. | | |
| | **COPD (journal summary)** | Supportive, palliative, and end-of-life care are beneficial for patients with advanced disease. (Main recommendation) | In patients with advanced COPD, palliative care services improve symptom control and manage psychosocial and spiritual concerns. | |

| | | | | |
|---|---|---|---|---|
| | **COPD (primary care summary)** | | | |
| | **Dementia (full)** | | | |
| | **Dementia (journal summary)** | | | |
| | **Dementia: deprescribing** | | | |
| | **Heart failure (full)** | The integration of palliative care into the multidisciplinary heart failure team is effective in …. (Rationale) | The benefits of a palliative care service should be considered in patients diagnosed with advanced heart failure. (Benefits and harm).<br><br>Palliative care services in the home were also effective in reducing rehospitalisation. | There are numerous benefits of collaborative care in primary care, provided pathways for communication are well established such as continuity of care and shared management plans. |
| | **Heart failure (summary)** | | | |

7
